# Characteristics associated with social anxiety in adults with developmental stuttering: A review

**DOI:** 10.18103/mra.v12i10.5876

**Published:** 2024-10-22

**Authors:** K.R. Bauerly

**Affiliations:** 1 University of Vermont

## Abstract

People who stutter are at a greater risk for developing symptoms of social anxiety, with up to 22–60% of adults who stutter meeting the criteria for a clinical diagnosis. Negative attitudes and feelings about speaking and stuttering are reported to emerge as early as the preschool years and are suspected to be due to exposure to negative listener reactions, stereotyping and social isolation. Repeated negative experiences lead to feelings of fear, embarrassment and loss of control during speaking which over time, leads to the development of more severe difficulties with speaking and an overall apprehension to speak as they perceive themselves as an incompetent communicator. The present review aims to summarize risk factors, particularly temperament and environmental factors, that are reported to play a role in the emergence and maintenance of social anxiety in people who stutter. Another aim of this review is to summarize the features of social anxiety reported in adults who stutter, some of which, are similar to high socially anxious fluent speakers (e.g., avoidant strategies) while others are specific to stuttering (e.g., muscle tension). The clinical implications of these findings and recommendations for future research are also discussed.

## Introduction

Developmental stuttering is a neurodevelopmental, early age onset disorder (DSM-IV Axis I) affecting approximately 1 % of the adult population^1,2^. Symptoms of stuttering typically emerge in preschool children between the ages of 2 and 3 years old. There is a strong tendency for children, particularly females, to recover from stuttering spontaneously, as the sex ratio of boys to girls is approximately 1.2 at onset while in adulthood the ratio increases to approximately 3:1^3^. Early signs of stuttering include overt breakages in the fluent flow of speech including part-word, syllable or word repetitions as well as prolongations, breakages in sounds, or hard glottal attacks^1^. As the disorder persists, the stutters often become more frequent and can be accompanied with tension and struggle^1,2,4^. At the same time, negative attitudes towards communication are found to emerge^5,6^ and are suspected to be due to exposure to negative listener reactions, stereotyping and social isolation^7,8^. For instance, research shows that preschool children show a preference to interact with their fluent peers^9^ and have been observed to tease ignore or interrupt a peer who stutters^10^. Children who stutter respond to these negative social interactions by reporting feelings of fear, embarrassment, shame and loss of control during speaking. Over time, the child who stutters begins to show more severe difficulties with communication and report apprehension to talk as they perceive oneself as an incompetent communicator^7,11,12^. In response to these struggles, escape behaviors begin to emerge in early childhood as an attempt to minimize a stuttering moment and may include visible tension or body movements such as head or hand jerking, eye blinking or sudden exhalation of the breath^1^. Avoidance behaviors are also observed to emerge as attempts to avoid a stuttering moment from occurring and may include word substitutions or circumlocutions, strategies to delay or prevent a stutter (e.g., “well”, “you know”), starting tricks (e.g., “uh”, “um”) and anti-expectancy behaviors (e.g., speaking with a rapid monotone)^1,4^.

People who stutter are at a higher risk for developing symptoms of social anxiety, with up to 22–60% of adults who stutter (AWS) meeting criteria for a clinical diagnosis^13–18^. The prevalence of social anxiety in AWS has been extensively studied using self-report measures such the Fear of Negative Evaluation^19^, Inventory of Interpersonal Situations^20^, Social Avoidance and Distress Scale^19^ and Social Phobia Anxiety lnventory^21^ . A meta-analysis by Craig & Tran^1^ reported substantially elevated social anxiety levels (effect size= .82) in AWS compared to controls. Kraaimaat et al.^22^ reported that AWS were significantly more likely to self-report higher on scores of emotional tension and discomfort with speaking and to report a significantly lower frequency of social responses compared to controls. In a later study, lverach et al.^23^ found in a group of 275 AWS, that the high socially anxious AWS (n=82) were more likely to report using avoidance behaviors and experience dissatisfaction with speaking and stuttering. Similar results were reported in Tomisato et al.^24^. Other studies have found high socially anxious AWS are more likely to interpret social situations negatively, report greater difficulties with daily communication and self-report greater stuttering severity^12,25^. Blood et al.^7^ found that self-reports of social anxiety, fear of negative evaluation and dissatisfaction with life are more likely to be experienced by AWS who experience childhood victimization, including physical, verbal, relational or cyber bullying.

## Risk factors for the development of social anxiety in people who stutter

Despite the strong evidence for social anxiety in adolescents and adults who stutter, we know very little about the risk factors leading to social anxiety during the childhood years. Several characteristics associated with social anxiety in children who do *not* stutter have been identified including temperament and environmental factors, both of which are reported in children who stutter.

## Temperamental factors

Temperament refers to biological based traits that include emotional, motor and attentional reactions as well as the self-regulatory responses to a situation^26,27^. Research shows that socially anxious children who do not stutter are reported as shy, quiet, reticent and show negative affectivity and low adaptability to uncertain or changing situations^28–31^. Also, socially anxious children are less likely to initiate or maintain an interaction and are often reported to show cautiousness, fear, and withdrawal from social situations and activities^29,32^. It is not surprising, therefore, to find that an inhibited temperament serves as a prominent risk factor to the development of social anxiety in childhood^33–38^.

Several of these temperamental risk factors to social anxiety reported in children who do not stutter have been reported in children who stutter^39–44^. Through parental reports and behavioral observations, studies have reported children who stutter to exhibit a more negative affect, lower self-regulatory abilities, are less adaptable to change, show increased emotional responses and are more behavioral inhibited^39–49^. For instance, Ntourou et al.^47^ and Choi et al.^48^ reported higher scores on the Short Behavioral Inhibition Scale in children who stutter compared to low anxious controls. Using a more direct measure of behavioral inhibition, the Go/NoGo task, Eggers et al.^41^ reported school age children who stutter to have lower inhibitory control compared to controls. Several studies have found associations between these temperamental characteristics and stuttering severity. For instance, Ntourou et al.^47^ showed a positive association between behavioral inhibition scores and stuttering severity, as well as self-reported speech attitudes. Tumanova et al.^43^ reported temperament scores were positively associated with the use of physical behaviors that accompany moments of stuttering. Frost^50^ also reported inhibited children who stutter are more likely to exhibit more secondary behaviors. While temperamental characteristics has been shown to be related to stuttering severity^49^ and the use of secondary behaviors,^50^ the direct relationship between temperament and social anxiety in children who stutter has received very little attention^51^.

## Environmental factors

Cognitive models of social anxiety propose that the relationship between temperament and social anxiety is further influenced by environmental factors^26,31,33^. While temperamental traits have been considered to be relatively stable and consistent across situations, more recent evidence suggests that temperament is responsive to environmental influences and therefore, may evolve overtime^30,32,52^. It is suggested that children with a behaviorally inhibited temperament are at greater risk for developing social anxiety if they also experience repeated, adverse environmental experiences. A number of environmental factors have been identified as possible risk factors to social anxiety including parenting style^32,53^, parental mental health^54–56^, and adverse life events^57,58^. There is evidence that an over-controlling parenting style, lack of warmth/rejection, and overprotection during the elementary years strengthens the relationship between temperament and social anxiety in adolescents ^26,33,59^. Other studies report that parents with social anxiety are significantly more likely to have a child with a social anxiety disorder^60^. Furthermore, a number of studies have linked stressful life events with the development of social anxiety such as changing schools, new sibling, divorce or family death^60^. Spence and Rapee^31,52^ propose that these environmental factors interact with temperamental characteristics and interfere with the child’s acquisition of social skills, interpersonal problems and emotional regulation abilities^53,61^.

The impact environmental factors can have on the interaction between temperament and social anxiety is particularly important for our understanding of childhood stuttering^1^. Several of the environmental risk factors for social anxiety identified in children who do not stutter have also been reported in children who stutter including parenting style^62^ and adverse life events^63–65^. For instance, parents of children who stutter are observed to be more demanding or anxious compared to parents of peers^66,64^. A retrospective study reported that children perceived their parents to be lower in attachment and reported frustration with their attempts to remediate their stuttering^62^. Also, a few studies have reported the onset of stuttering following a traumatic life event^63^, while others found no relationship^67^. However, these studies have only assessed the effects of environmental factors on the onset of stuttering, leaving the impact of social anxiety unexplored. Children who stutter with a more inhibited temperament are particularly vulnerable to negative social experiences as their increased emotional reactivity^42^ coupled with a decrease in adaptability and emotional regulatory abilities^68^_·,_ puts them at a greater risk for developing symptoms of social anxiety^52,58^. As social behavior is learned through experiences, these children who stutter will have lower social confidence and poorer communication skills^40,43^. Over time, high socially anxious children who stutter are reported to fear negative listener reactions^10^ and begin to anticipate a stuttering movement and in response, begin to develop secondary behaviors as an attempt escape or avoid a stuttering moment.

## Characteristics of social anxiety disorder in people who stutter

### FEAR OF NEGATIVE EVALUATION

One important characteristic of social anxiety is the fear of being negatively evaluated. Fear of negative evaluation is defined as an apprehension about others’ evaluation^19^. High socially anxious speakers are more likely to interpret a listener’s emotional expression as negative and as such, lead to anticipating others will evaluate themselves negatively as well^69^. Fear of negative evaluation is most commonly measured using the self-report, Fear of Negative Evaluation^19^ or the abbreviated Brief Fear of Negative Evaluation^66^. Studies report that high socially anxious speakers experience anxiety when evaluating previous social interactions and anticipating future ones^70^. These negative feelings are found to lead to feelings of worry, dread and anxiousness as well as sweating and rapid heart rate^71–73^. Fear of negative evaluation is found to predict social anxiety levels^72^.

Adults who stutter (AWS) have self-reported significantly greater levels of fear of evaluation compared to adults who do not stutter (ANS) using the Brief Fear of Negative Evaluation (BFE)^7,15,18,66^. Increases in negative social experiences can contribute to the onset and maintenance of fear of negative evaluation as well as the emergence of safety behaviors^74,75^. For instance, in a group of 133 AWS, Lowe et al.^74^ reported a significant association between the use of safety behaviors (e.g., avoiding difficult words or socially threatening situations) and self-report measures of fear of negative evaluation^19^ and Unhelpful Thoughts and Beliefs about Stuttering (UBSTAS)^76^. However, others have reported no significant differences in avoidance behaviors between AWS and individuals with a diagnosis of social phobia^16^. These discrepancies may be due to individual differences among AWS^16,77,78^. For instance, Brundage et al.^79^ found no group differences between AWS and a group of controls on the Fear of Negative Evaluation questionnairre^66^; however, after forming subgroups based on high versus low scores, they found the AWS who scored the highest on the Fear of Negative Evaluation also scored significantly higher on an Interpretation and Judgement Questionnaire^80^, which is a questionnaire that quantifies negative interpretation and judgment biases for 24 social situations ranging from negative (e.g., *“A friend tells you that a colleague dislikes you”* to ambiguous (e.g., *“The newly introduced person doesn’t say anything to you”)*.

### ANTICIPATORY PROCESSING

Over time, the AWS begins to anticipate negative social experiences prior to speaking^81–83^, which promotes the use of safety behaviors as an attempt to avoid or escape from a potential stuttering moment^4,74^. Prior to a social situation, high socially anxious speakers are reported to imagine in detail what might happen during a social event which elicits further anxiety and dread about the up-and-coming situation^84–86^. These cognitive processes often lead to completely avoiding the situation^1^, which perpetuates and maintains anxiety over the long-term. Similar effects have been reported in AWS. Anticipating a stuttering moment is a common phenomenon reported in AWS and is defined as a covert experience of anticipating an embarrassing moment will take place^83,87,88^. Jackson et al.^88,89^ describes this as long term anticipation where the AWS ruminates over an up- and- coming speaking situation (e.g., work meeting, social gathering) which subsequently elicits the engagement of self-management strategies such as previewing speech, changing speech rate, employing fluent enhancing techniques (e.g., easy onset) or avoiding the situation all together^88^. Jackson et al.^88^ reported that up to 77% of AWS experience anticipation “often” and that these anticipatory events are not related to the AWS’ stuttering severity or treatment history. As described in Jackson^81^, AWS are also reported to undergo immediate or short-term anticipation where a stutter is anticipated to occur in the moment of speaking and is suspected to be associated in time with negative thoughts and autonomic arousal^81,90^. Using open-ended questions, Jackson et al.^88^ found anticipating a stutter occurs at a cognitive level (e.g., negative thoughts) and is associated with learned negative fears to sounds and/or words. As such, anticipating a stuttering moment can lead to transient moments of anxiety, muscle tension, and/or avoidant strategies ^5,81,88^ as well as sympathetic nervous system increases^90^. Avoidant strategies include replacing or circumlocution around a word they suspect will be stuttered or avoiding a speaking situation all together. Jackson et al.^88^ reported that some AWS felt that anticipation was helpful as it allotted them time to prepare for a self-management strategy such as relaxing their muscles or employing a fluency skill, while others reported to respond to the anticipation of stuttering with fear or dread.

### AUTONOMIC REACTIVITY AND REGULATORY RESPONSES

A number of studies have used objective measures of sympathetic and parasympathetic indices to measure anxiety–related changes during speaking. The sympathetic and parasympathetic branches of the autonomic nervous system work complementary to one another to help regulate the day-to-day changes in emotional responses to internal and external demands^91–93.^ The sympathetic nervous system or “fight or flight” function, prepares the body for stress by eliciting increases in heart rate and breathing as well as sweating of the eccrine glands^93,94^. Skin conductance levels (SCL) is one of the most extensively used for measuring sympathetic activity ^91,93^. It is a tonic measure of electrodermal activity which reflects eccrine sweat gland activity controlled by the sympathetic nervous system^94,95^. Increases in sympathetic nervous system activity occur when the autonomic fibers from the vagus nerve, sending regulatory signals to the heart, lungs and digestive organs, are inhibited^96^. When the vagus nerve is disinhibited, parasympathetic influences dominate, resulting in decreases in heart rate, slower breathing and decreases in sweat production. Parasympathetic nervous system activity can be indexed by measuring respiratory sinus arrhythmia (RSA), which is a metric of high frequency HR variability. (i.e., beat-to-beat variability). An increase in RSA (i.e., increase in inhibitory control on the sympathetic branch) is associated with decreases in heart rate and increases in HR variability^93^.

The reciprocal relationship between the sympathetic and parasympathetic nervous system in response to social stress has been widely studied^97,98^. Studies report high socially anxious speakers exhibit elevated levels of sympathetic activity and subsequent lower parasympathetic influences at rest and during socially stressful speaking tasks^94,99^. Research has shown that increases in RSA reflect positive affect^100^ as well as improved behavioral regulation^101^, social engagement^98,102,103^ and effortful control (i.e., regulation of appetitive or aversive stimuli) ^104,105^. For instance, by tracking facial expressions and RSA levels when viewing negative stimuli, Pu et al.^106^ reported that individuals with high RSA levels were better able to suppress negative emotion in a nonclinical population. Others report high RSA in individuals who are more effective at regulating stress through the use of attentional processes^107^ and self-control strategies ^106,108^ such as cognitive reappraisal or suppression^109,110^. From this perspective, increased RSA (i.e. vagal input) may help prevent or reduce daily responses to stress^109^ and improve the ability to socially engage in a flexible, adaptive manner^96^. Conversely, lower RSA levels or decreased heart rate variability, has been found in high anxious individuals and has been suggested to contribute to poor inhibitory control and reduced attentional regulation^111–113^. Importantly, not all studies report increased sympathetic and decreased parasympathetic responses in high anxious speakers. Some studies report decreases in cortisol levels and sweat production subsequent to reduced heart rate and increased RSA levels in high anxious speakers and suggest this is a defensive response, due the need for increased attention and hypervigilance of their surroundings^98,114^ An increase in RSA, however, renders the autonomic nervous system less able to respond effectively to environmental stimuli^98^.

Heightened sympathetic nervous system activity has been reported in both children and adults who stutter^115–117^ . Zimmerman^118^ suggested that increases in sympathetic activity in response to emotional arousal is involved in the motoric breakdown leading to disfluencies. Since then, research has found increased sympathetic activity in AWS when anticipating or in response to a stressful speaking task^115,119,120^. Bowers et al.^115^ reported significant increases in SCL in AWS when anticipating a feared versus neutral word and decreases in SCL when speaking in a fluency promoting condition (i.e., choral reading). Dietrich & Roamen^119^ reported significant increases in SCL in AWS before and during a self-identified fearful speaking task. Other studies have reported similar increases in SCL in AWS during socially stressful tasks such as giving a job interview^120^ and speaking in front of an audience^121^. On the contrary, several studies have reported no significant differences in sympathetic arousal between AWS and ANS during speaking tasks^122–126^. Studies assessing children who stutter during emotionally driven conditions have more consistently reported higher SCL during a range of speaking tasks. For instance, Zengin-Bolatkale et al.^117^ reported significantly higher SCL in children who stutter compared to children who do not stutter while performing a cognitively stressful task. However, these group differences diminished with age. Jones et al.^116^ reported higher SCL in children who stutter versus children who do not stutter while viewing positively and negatively valanced video clips while the group of controls exhibited higher SCL when viewing only the negatively valanced video clip. However, Tumanova et al.^127^ reported no differences in emotional reactivity between children who stutter versus children who do not stutter. It is possible that over time the AWS learns to develop coping strategies that reduce the emotional reactivity to emotionally arousing situations.

Few studies have assessed the emotional regulatory abilities in AWS^124,128^. Bauerly et al.^124^ reported significantly higher RSA levels in AWS compared to ANS during rest and when preparing to give a speech. AWS’ increased in RSA levels were suspected to be due to a need to continuously engage in an emotional regulatory strategy. A later study revealed that AWS’ self-reports of trait and social anxiety were predictive of RSA levels during resting conditions^128^. That is, those who scored the lowest on self-reports of trait (i.e., STAI-T^129^) and social anxiety (i.e., SIAS^21^) were the ones who showed the highest RSA levels. The authors interpreted these findings to suggest that low self-reported anxiety scores simultaneous with high RSA levels in some AWS may reflect a self-regulatory strategy adopted in response to the chronic stress associated with stuttering.

While it is clear that feelings of social evaluative stress can lead to a set of psychological and physiological responses, studies have shown that these two stress responses do not always co-occur^130,131^. In support, Brundage et al.^126^ reported no significant differences in skin conductance levels or heart rate in AWS across low- and high socially stressful speaking situations; however, subjective ratings of stress were significantly higher when speaking under high social stress. Similarly, Bauerly & Bilardello^132^ reported no significant relationship between SCL and self-reports of anxiety in AWS. While research in this area is limited, results suggest that psychological and physiological stress responses may be governed by additional processes and one area receiving considerable amount of attention in psychology is the relationship between social stress responses and attentional processing^125,133,134^.

### ATTENTIONAL PROCESSING IN RESPONSE TO SOCIAL STRESS

Anxiety theories have suggested that an internal attentional focus to physiological reactions (e.g., increase in sweating) and/or negative thoughts (e.g., embarrassment) may exacerbate anxiety levels^130,135^. This line of research suggests that an increase in perceived levels of anxiety (i.e., psychological response) may be due to a tendency to focus their attention inward, on the self, when speaking in a socially stressful situation which is suspected to be an attempt to regulate heightened emotions^84,94,95^. However, these attentional shifts are not without consequences. Research shows that maladaptive attentional processing may lead to increases in negative, self-conscious thoughts (e.g., dread, embarrassment, shame), causing an increase in perceived levels of anxiety. Also, this self-focused attention can impair the speaker’s ability to attend to the listener and receive social feedback, leading to disruptions in social interactions^135,136^.

While some studies have shown AWS to exhibit a hyperawareness to negative listener faces when anticipating a socially stressful speaking condition^74,125,137^, others have reported a shift away from listener reactions during the moment of speaking. For instance, when giving a speech to an audience, Lowe et al.^78^ found AWS attended less to audience members, regardless of facial expressions, and more time on the background. When comparing the attentional focus across different emotional faces in the audience, the AWS looked for less time at audience members depicting positive faces and more time on audience members showing negative and neutral faces. Further analysis revealed that the avoidance of positive faces was associated with negative subjective ratings of performance and increased perceived levels of anxiety. Several other studies report that adolescents and adults who stutter focus their attention inward during socially stressful situations. That is, they focus on anxiety-related symptoms such as physiological (e.g., heart rate, sweating) and psychological changes (e.g., negative thoughts)^5,138–141^. These attentional shifts are suggested to be a strategy used to regulate emotions; however, it comes with a cost as this results in an interference in communication as the speaker is not benefiting from positive listener reactions and may be perceived by the listener as uninterested in the conversation.

### INTERPRETATION BIASES IN RESPONSE TO SOCIAL STRESS

Social interactions involve interpreting listener reactions which can range from positive (e.g., smiling), negative (e.g., furrowed eyebrows), or ambiguous (e.g., looking at watch). Research shows that socially anxious speakers are more likely to interpret social information as negative and lack positive interpretations of social cues (e.g.,^133,142–146^). For instance, a yawn may be interpreted by a high socially anxious speaker as boredom (i.e., negative) compared to tired (i.e., neutral). Cognitive models of social anxiety^84^ propose that frequently interpreting ambiguous information as negative heightens and maintains anxiety. While Chen et al.^133^ reported large effects sizes for both the clinical and subclinical populations, not all studies report negative biases in socially anxious speakers which they suspect may be due to methodological differences. In Chen et al.’s review, strongest effect sizes were reported in studies that employed subjective, self-report measures or interpretation questions immediately following a social scenario^147,148^. However, objective measures (e.g., reaction time studies), where participants respond to verbal (e.g., written scenarios) or visual (e.g., photographs, video scenarios) stimuli have also yielded strong relationships^149–152^.

Negative interpretation biases were first reported in AWS by Brundage et al.^153^ using a negative judgement bias questionnaire and later Rodgers et al.^154^ found high socially anxious adolescents who stutter to exhibit ambiguous social vignettes as negative. Research assessing AWS’ preferential tendency to interpret negative meanings from ambiguous social cues is in need of further research. lnterpretating ambiguous information as negative may be especially harmful to AWS as social cues are often ambiguous (e.g., smiling) but can easily be interpreted as threatening (e.g., *“I look stupid”)* as opposed to benign (e.g., *“They enjoy my company”)* and as such, exasperate anxiety and lead to an increase in safety behaviors and stuttering severity^154^.

In summary, social anxiety is frequently reported in both children and adults who stutter. Responses to socially threatful situations begin as early as preschool and may result in the elicitation of negative thoughts and feelings about speaking, facial tension and physical struggle as well as the use of escape (e.g., physical movements) and avoidance (e.g., circumlocutions) behaviors. These behaviors cause the disorder to worsen and become more complicated to treat. Evidence for social anxiety using self-reports is strong, while objective measures are less consistent, suggesting that the emergence and maintenance of social anxiety in AWS is more cognitive driven. Studies of adolescents and adults who stutter have reported both attentional and interpretation processing biases, which are suspected to lead to the long-term maintenance of anxiety symptoms and further disruptions in communication, thus perpetuating the cycle of anxiety.

## Recommendation for future research

Research is needed into the identification of risk factors for social anxiety in people who stutter and determining whether these symptoms lead to the subsequent use of maladaptive, safety behaviors. Developing tools to enable clinicians to identify those children who are at risk for social anxiety will facilitate more individualized treatment approaches. Traditional stuttering treatment programs focus on replacing stuttered speech with a novel speech pattern that promotes fluency (e.g. easy onset). While immediate fluency gains are reported with this type of approach, there is a high rate of relapse^1^. The lack of long-term maintenance is likely due to the failure of these programs to go beyond the AWS’ motoric disability and address symptoms of social anxiety. There are several different therapeutic approaches and techniques to addressing the cognitive and social aspects related to social anxiety and stuttering. Many of them incorporate basic strategies for addressing negative cognitions related to speaking and stuttering including cognitive restructuring, exposure or desensitization and attentional training^75,155–157^. For instance, the negative cognitions associated with stuttering are addressed using Cognitive Behavioral Therapy (CBT) for stuttering where problem-solving methods are used to identify distressful feelings and thoughts about speaking and modify them to promote increased participation and positive self-perceptions^156,158^. Several programs aim to reduce the frustration that emerges from stuttering through acceptance and mindfulness techniques^157,159^; while other programs aim to reduce escape and avoidance behaviors as well as emotional reactivity to moments of disfluency with the goal of achieving struggle-free, forward-moving disfluencies^155^ . For instance, both CBT for stuttering158 and Avoidance Reduction Therapy for Stuttering (ARTS^®^);^155^ encourage the speaker to expose themselves as a person who stutters by reducing avoidance behaviors and openly stuttering in an attempt to reduce their fearful thoughts about speaking. While these therapy programs differ in their therapeutic approaches and techniques, they all incorporate basic strategies for addressing the social, emotional, and cognitive issues often associated with stuttering^158^. Considering that AWS are at a significant risk for developing symptoms of social anxiety^77^, it is not until treatment begins to address symptoms related to social anxiety will we see long-lasting changes from therapy^158^. Future research looking more closely at the maladaptive behaviors that accompany social anxiety in AWS, particularly interpretation and attentional biases, is needed in order to facilitate further improvements in treatment.

## Conclusion

Adults who stutter are at greater risk for developing social anxiety. Symptoms of social anxiety begin to emerge early, as young as 3 years old. While research is needed in determining risk factors for social anxiety in this population, current evidence suggests that temperament and environmental factors play a role in the emergence and maintenance of symptoms associated with social anxiety. Several features of social anxiety found in fluent speakers are also found in adults who stutter, including fear of negative evaluation, maladaptive anticipatory and autonomic processing as well as information processing biases. Other symptoms such as the adoption of secondary, safety behaviors (e.g., escape behaviors) are more specific to the disorder of stuttering. Developing tools to enable clinicians to identify the behaviors associated with social anxiety in people who stutter will facilitate more individualized treatment programs targeting the reduction of social anxiety and the facilitation of long term treatment outcome.
